# Does past/current pain change pain experience? Comparing self-reports and pupillary responses

**DOI:** 10.3389/fpsyg.2023.1094903

**Published:** 2023-02-17

**Authors:** Hyunkyung Yoo, Younhee Cho, Sungkun Cho

**Affiliations:** Department of Psychology, Chungnam National University, Daejeon, Republic of Korea

**Keywords:** pain, past pain experience, current pain experience, reappraisal, self-report, pupillary response

## Abstract

**Introduction:**

For decades, a substantial body of research has confirmed the subjective nature of pain. Subjectivity seems to be integrated into the concept of pain but is often confined to self-reported pain. Although it seems likely that past and current pain experiences would interact and influence subjective pain reports, the influence of these factors has not been investigated in the context of physiological pain. The current study focused on exploring the influence of past/current pain on self-reporting and pupillary responses to pain.

**Methods:**

Overall, 47 participants were divided into two groups, a 4°C–10°C group (experiencing major pain first) and a 10°C–4°C group (experiencing minor pain first), and performed cold pressor tasks (CPT) twice for 30 s each. During the two rounds of CPT, participants reported their pain intensity, and their pupillary responses were measured. Subsequently, they reappraised their pain ratings in the first CPT session.

**Results:**

Self-reported pain showed a significant difference (4°C–10°C: *p* = 0.045; 10°C–4°C: *p* < 0.001) in the rating of cold pain stimuli in both groups, and this gap was higher in the 10°C–4°C group than in the 4°C–10°C group. In terms of pupillary response, the 4°C–10°C group exhibited a significant difference in pupil diameter, whereas this was marginally significant in the 10°C–4°C group (4°C–10°C: *p* < 0.001; 10°C–4°C: *p* = 0.062). There were no significant changes in self-reported pain after reappraisal in either group.

**Discussion:**

The findings of the current study confirmed that subjective and physiological responses to pain can be altered by previous experiences of pain.

## 1. Introduction

Pain is defined as “an aversive sensory and emotional experience typically caused by, or resembling that caused by, actual or potential tissue injury” (Raja et al., 2020). As noted in this definition, pain is not merely nociception but is an intrinsically complex inner experience consisting of a biopsychosocial process, which could vary according to biological, emotional, cognitive, and contextual factors (Love-Jones, 2019). One factor that influences pain experience is past pain experiences, which are related to the biological and psychosocial responses to pain (Rollman et al., 2004). For example, patients who had experienced pain in the past reported higher pain sensitivity than patients who had a pain-free experience, even if they no longer experienced pain at the moment (Phillips et al., 2022). Additionally, past pain experiences that were highly intense contribute to high reactivity to pain (Rollman et al., 2004). The placebo analgesic effect is altered by past pain experiences as well (Geers et al., 2015). Another factor that affects pain is the current state of pain. When people recall previous pain experiences, their intensity is estimated based on the current pain experience (Haas et al., 2002; Bąbel et al., 2018). In addition, by assimilating it, current pain impacts the perception of past pain (Eich et al., 1985).

It is likely that past and current pain interact with each other and influence the pain experience. However, the relationship between past and current pain experiences has only been investigated in self-reported pain. Since pain is a subjective experience, the most widely used method to assess pain is single-item pain scales such as the visual analog scale (VAS) or the numeric rating scale (NRS) (Hjermstad et al., 2011). Despite these single-item scales showing promising psychometric properties (Bijur et al., 2001; Goddard et al., 2004; Begum and Hossain, 2019), self-reporting pain scales are prone to bias owing to the arbitrary interpretation of self-reporting pain scales (Williams et al., 2000; Tracy, 2017; Johannessen, 2019) and temporal changes (DeLoach et al., 1998; Leino et al., 2011). In addition, the usefulness of these scales is confined to low/high levels of pain due to ceiling and floor effects, making it difficult to measure moderate levels of pain by influencing their reliability and interpretation (Bijur et al., 2001; González-Fernández et al., 2014). Furthermore, because self-reporting scales share considerable variance with factors other than pain (e.g., pain interference and emotional aspects) (Thong et al., 2018), it is unclear which aspects of pain they truly measure. Considering the limitations mentioned above, self-report scales are not precise enough to capture the dynamics of a pain experience influenced by past/current pain, and this leaves some points to be filled in by other ways of measuring pain.

To design an elaborate interpretation of a pain experience, pain-related physiological responses have been suggested as a parameter that allows for the quantification of pain intensity (Tracey et al., 2019). There is an apparent association between nociception and the autonomic nervous system (ANS) (Benarroch, 2006), and it is possible to discriminate between noxious and non-noxious stimuli from ANS parameters to some extent (Ben-Israel et al., 2013). Therefore, many previous studies have attempted to overcome the limitations of self-reporting using ANS parameters (Lin et al., 2018; Subramaniam et al., 2018; Jiang et al., 2019). As the pupillary response is linked to both the sympathetic and parasympathetic nervous systems (Bouffard, 2019), it has been suggested as a useful and informative parameter for ANS activity caused by pain (Cowen et al., 2015; Charier et al., 2017). Furthermore, the pupillary response is coupled with the activity of the locus coeruleus (LC), the brain region involved in the neuromodulation of the noradrenergic system (Larsen and Waters, 2018). The processing and modulation of pain are also involved in the LC (Suárez-Pereira et al., 2021). In addition, LC-mediated stress responses caused by pain can therefore be inferred through the pupillary response (Szabadi, 2012).

Although many studies utilizing pupillary response have confirmed its practicability as a proxy of nociception, these studies have focused on reducing bias and error in measuring the pain experience but overlooked other dimensions that impact the overall pain experience (Charier et al., 2017; Wang et al., 2022). For a comprehensive understanding of the pain experience, it is necessary to move beyond merely pursuing an “objective” pain measurement. To gain a better understanding of the subjective nature of pain, it is plausible to consider a physiological response to pain as an observable proxy, which also reflects the emotional, cognitive, and contextual perspectives of the pain experience. Given the multidimensional aspects of pain, the influence of contextual factors such as past/current pain has yet to be extensively investigated, considering physiological pain as just one component of the total subjective pain experience. By elucidating how the pain response changed in the subjective perception and physiological pain experience depending on the past and current pain experience, it should be possible to gain a better understanding of pain experience.

Motivated by this background, this study aimed to investigate the influence of past and current pain by recording self-reported pain and pupillary responses. We manipulated past and current pain experiences using a cold pressor task (CPT) with different intensities of cold pain stimuli. We then compared self-reported and pupillary responses to cold pain stimuli with different experiences of past and current pain. Additionally, we examined whether the appraisal of previous pain could be affected by the current pain experience. Using this approach, we attempted to demonstrate how past pain influences the current pain response, and how the perception of previous pain varies with the current state of pain. Based on previous findings, we hypothesized the following: 1) both self-reported pain and pupil diameter increase as participants experienced longer CPT and both indices would be higher in the 4°C CPT than in the 10°C CPT regardless of the group; 2) in the second CPT, participants who experienced 4°C in the first CPT will report higher pain intensity compared with their counterparts who experienced 10°C in the first CPT, even though they conducted the second CPT at a higher temperature (10°C) than the first CPT (4°C); 3) in the second CPT, pupil diameter will be larger in the 4°C CPT than 10°C CPT irrespective of the temperature of the first CPT; and 4) participants who experience 4°C in the second CPT will downgrade their past pain intensity compared with the participants who experience 10°C in the second CPT.

## 2. Methods

### 2.1. Participants

**Figure 1:**
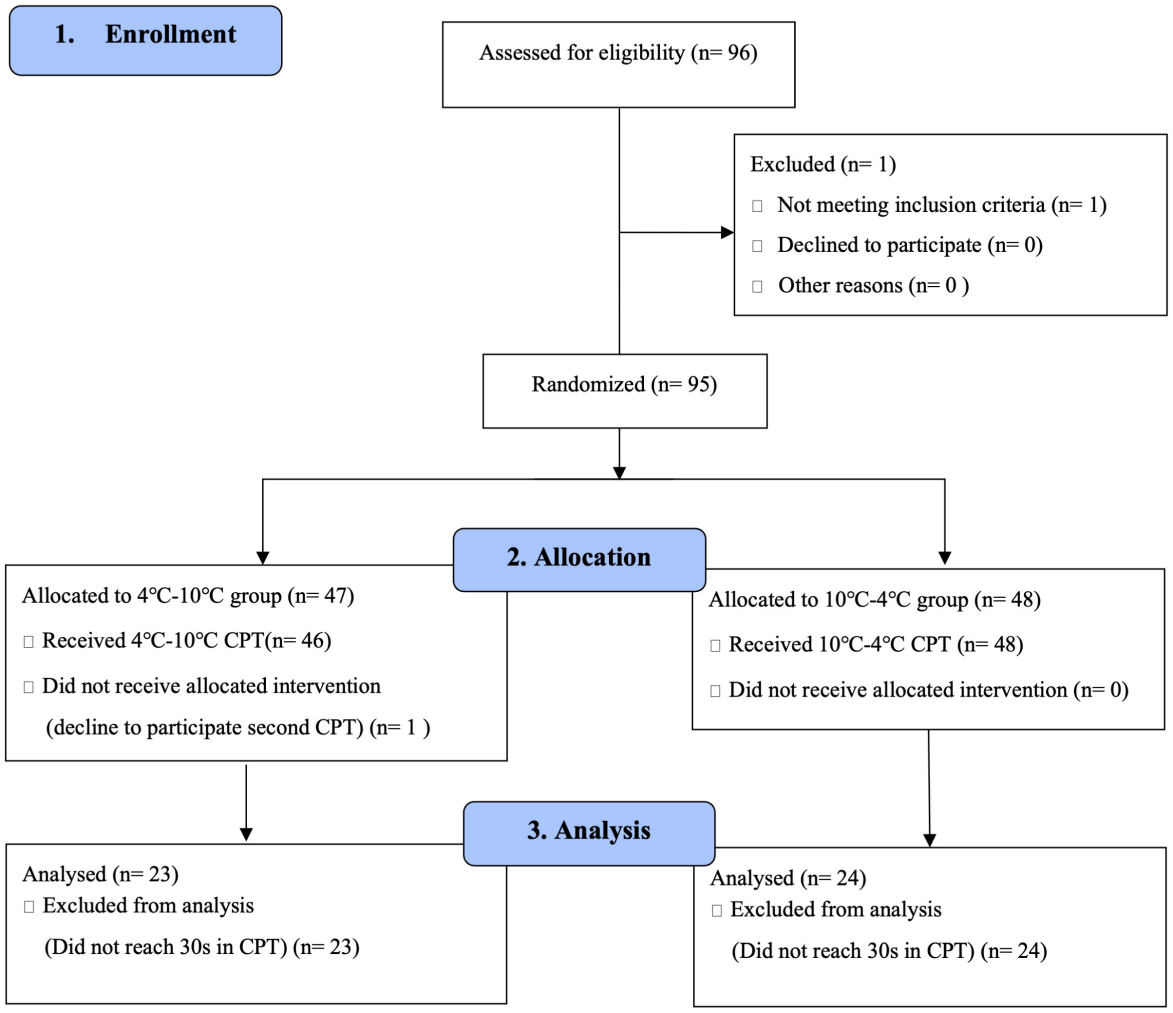
CONSORT flowchart of this study.

The participants were recruited from Chungnam National University through a university bulletin board between August 2020 and October 2021. Eligibility criteria were as follows: (1) age over 19 years; (2) never experienced CPT; (3) no substantial pain at baseline (no current pain >3/10 on the VAS when participants arrived at the laboratory); (4) no dermatological problems in the non-dominant hand (e.g., injury on hand); (5) without severe acute pain (e.g., traumatic injury, surgical treatment) or chronic pain history (no experienced pain>3/10 on VAS for more than 3 months, e.g., arthritis, chronic low back pain); (6) no ophthalmological problems (e.g., glaucoma); (7) no neuropsychological/psychiatric problems; (8) no recent use of analgesics; and (9) fluent in the Korean language. A power analysis was conducted using G*power software, version 3.1 (Faul et al., 2007). Based on the power analysis, at least 52 participants for the total sample size were recommended to detect an effect size of 0.20 with a power of 0.80 (α = 0.05). A total of 96 potential participants were enrolled, most of whom were undergraduate students. One participant was excluded because of the recent use of analgesics. Participants were randomly assigned to the 4°C–10°C (n = 47) and 10°C–4°C (n = 48) groups. Ninety-five participants participated in two rounds of CPT. Participants who tolerated CPT for over 30 s were included in the statistical analysis. Of the 95 participants, 47 did not meet this criterion for statistical analysis. The final sample consisted of 47 participants (group 4°C–10°C, n = 23; group 10°C–4°C, n = 24). Another participant was excluded because of declining participation in the CPT. All participants were compensated $8.50. See Figure 1 for the CONSORT flowchart.

### 2.2. Measures and apparatus

#### 2.2.1. Pain intensity

Pain intensity was measured using the 11-point NRS [i.e., 0, no pain at all; 10, worst pain imaginable (Jensen and Karoly, 2011)]. Therefore, a high score means that the participant experienced high pain intensity.

#### 2.2.2. Pain catastrophizing

Pain catastrophizing was measured using the Korean version of the Pain Catastrophizing Scale (K-PCS) (Cho et al., 2013). The K-PCS is a self-report questionnaire consisting of 13 items measuring pain catastrophizing (Sullivan et al., 1995). Each item is rated on a 5-point scale (0 = not at all to 4 = all the time). The total score ranged from 0 to 52, with a high score indicating high pain catastrophizing. The internal consistency of K-PCS was good (Cronbach’s α = 0.88).

#### 2.2.3. Pain anxiety

Pain anxiety was measured using the Korean version of the Pain Anxiety Symptom Scale (KPASS-20) (Cho et al., 2010). The KPASS-20 is a self-reporting questionnaire consisting of 20 items measuring pain-related anxiety symptoms (McCracken et al., 1992). Each item is rated on a 6-point scale (0 = never, 5 = always). The total score ranges from 0 to 100, with a high score representing high pain and anxiety. The internal consistency of the KPASS-20 is good (Cronbach’s α = 0.88).

#### 2.2.4. Positive affect and negative affect

Positive and negative affect were measured using the Korean version of the Positive and Negative Affect Schedule (PANAS) (Lim et al., 2010). The PANAS is a self-reporting questionnaire that consists of 10 items measuring positive mood and 10 items measuring negative mood (Schmukle et al., 2002). Each item is rated on a 5-point scale (1 = not at all to 5 = extremely). The total score ranges from 10 to 50 for each subscale. A higher score represents people experiencing a higher level of both positive and negative affects. The internal consistency of the positive affect subscale was good (Cronbach’s α = 0.84). For the negative affect subscale, the internal consistency was acceptable (Cronbach’s α = 0.70).

#### 2.2.5. Cold pressor task

CPT has also been used to induce experimental pain (Mitchell et al., 2004). CPT was conducted using a refrigeration tank (SH-WB-11R-OM) with a water circulation system manufactured by SAMHEUNG ENERGY, Korea. Before beginning, the participants were notified of the overall procedure. To control the baseline hand temperature, participants were asked to immerse their non-dominant hand in a plastic bath filled with warm water (36°C ± 0.5°C) for 2 min. After 2 min, the participants were required to put their hands into a tank filled with cold water maintained at 4°C or 10°C. The participants were blinded to the water temperature. Participants were allowed to withdraw their hand when they felt that they could no longer endure the pain. The upper time limit of CPT immersion was 30 s.

#### 2.2.6. Pupillary response

The pupillary response was measured using a monocular handheld digital infrared pupillometer (PLR™-3000) manufactured by NeurOptics (CA, United States). A pupillometer was used with an eye cup to reduce the impact of ambient light during measurement. The measurements were performed in a quiet room with an illuminance of 795 ± 5 lx. The participants’ right eye was exposed to a white light stimulus of 50 μW to control baseline pupil size, followed by 10 μW light stimulus during measurement, with pupillary response automatically recorded by the pupillometer. The measurement was completed 5 s after the participants withdrew their hands from the cold water.

### 2.3. Procedure

This study was approved by the Institutional Review Board of Chungnam National University (No. 202001-SB-009-01). Upon arrival at the laboratory at Chungnam National University, participants were randomly assigned to two groups (the 4°C–10°C group or the 10°C–4°C group). Participants then completed informed consent and self-reporting questionnaires (demographic information, K-PCS, KPASS-20, and PANAS). Subsequently, the participants performed the first CPT. Participants reported pain intensity every 10 s, and pupillary responses were measured during the CPT (assessment 1). Participants then took a 15-min break (recovery phase). In doing so, the participants were able to recover from CPT-induced pain. If a participant felt pain even after 15 min, they could take additional time for recovery. The second CPT was then carried out with a self-reported pain rating and pupillary response measurement (assessment 2). The overall pain intensity during CPT was reported at the end of every CPT. The temperature of CPT in assessments 1 and 2 was contingent on the group. The participants who were allocated to the 4°C–10°C group performed the 4°C CPT in assessment 1 and the 10°C CPT in assessment 2. Inversely, participants in the 10°C–4°C group performed the 10°C CPT first and the 4°C CPT later. Subsequently, all participants were asked to reappraise their pain intensity in the first CPT (reappraisal phase). In the reappraisal phase, the 4°C–10°C group reported pain intensity of 4°C CPT and the 10°C–4°C group reported pain intensity of the 10°C CPT. Finally, participants were thoroughly debriefed and compensated.

### 2.4. Statistical analysis

Data reduction and statistical analyses were conducted using SPSS 26 and R version 3.5.2. Independent *t-tests* and *χ^2^* tests were used to compare between-group differences at the baseline. As a primary analysis to investigate the effect of the group on outcome variables, a three-way (Group × Time × Temperature) mixed-measures analysis of variance (ANOVA) was applied. A significant three-way interaction was decomposed using a simple interaction analysis. A simple main analysis was performed if a simple interaction effect showed a significant result. A simple main analysis was used for two-way significant interaction effects. The pupil diameter index was calculated based on a previously described method (Eisenach et al., 2017). Pupil diameters of <20 mm and > 90 mm were considered outliers. Outliers were replaced with the mean pupil diameter of each individual. Data from 1 s to 35 s were included in the analysis. Pain ratings by NRS at each time point were used to assess the pain intensity. Additionally, we explored whether recent pain experiences influenced the rating of previous pain experiences. To compare the reappraised pain rating differences between groups, a two-way (Group × Measurement point) mixed-measures ANOVA was used. All tests were 2-tailed, and *p* < 0.05 of statistical significance criteria. The results are reported as the mean ± standard deviation in the tables and the mean ± standard error in the figures. The generalized eta squared (ŋ_G_^2^) was used to calculate the effect size (Bakeman, 2005).

## 3. Results

### 3.1. Descriptive statistics

The participant characteristics are summarized in Table 1. There was no significant difference between the groups in terms of age (*t*(45) = −1.21, *p* = 0.24) or gender (*χ^2^*(1) = 0.20, *p* = 0.77). Pain at baseline (*t*(45) = −0.83, *p* = 0.41), pain catastrophizing (*t*(45) = 0.05, *p* = 0.96), pain anxiety (*t*(45) = −5.41, *p* = 0.59), positive affect (*t*(45) = −0.18, *p* = 0.86), and negative affect (*t*(45) = −0.55, *p* = 0.59) showed no significant difference between the two groups.

**Table 1:** Descriptive statistics and group difference for baseline measures.

	Condition			
	4°C–10°C (*n* = 23)		10°C–4°C (*n* = 24)	
	*M*	*SD*	*M*	*SD*
Age	23.22	2.24	25.13	7.27
Gender *N* (%)	12 Female (52.20%)		11 Female (45.80%)	
Pain intensity (baseline)	0.57	0.73	0.83	1.37
Pain catastrophizing	13.83	9.55	13.71	7.29
Pain anxiety	36.74	13.63	39.29	18.27
Positive affect	29.78	7.81	30.17	6.56
Negative affect	17.57	4.21	18.33	5.30

### 3.2. Pain intensity

**Figure 2:**
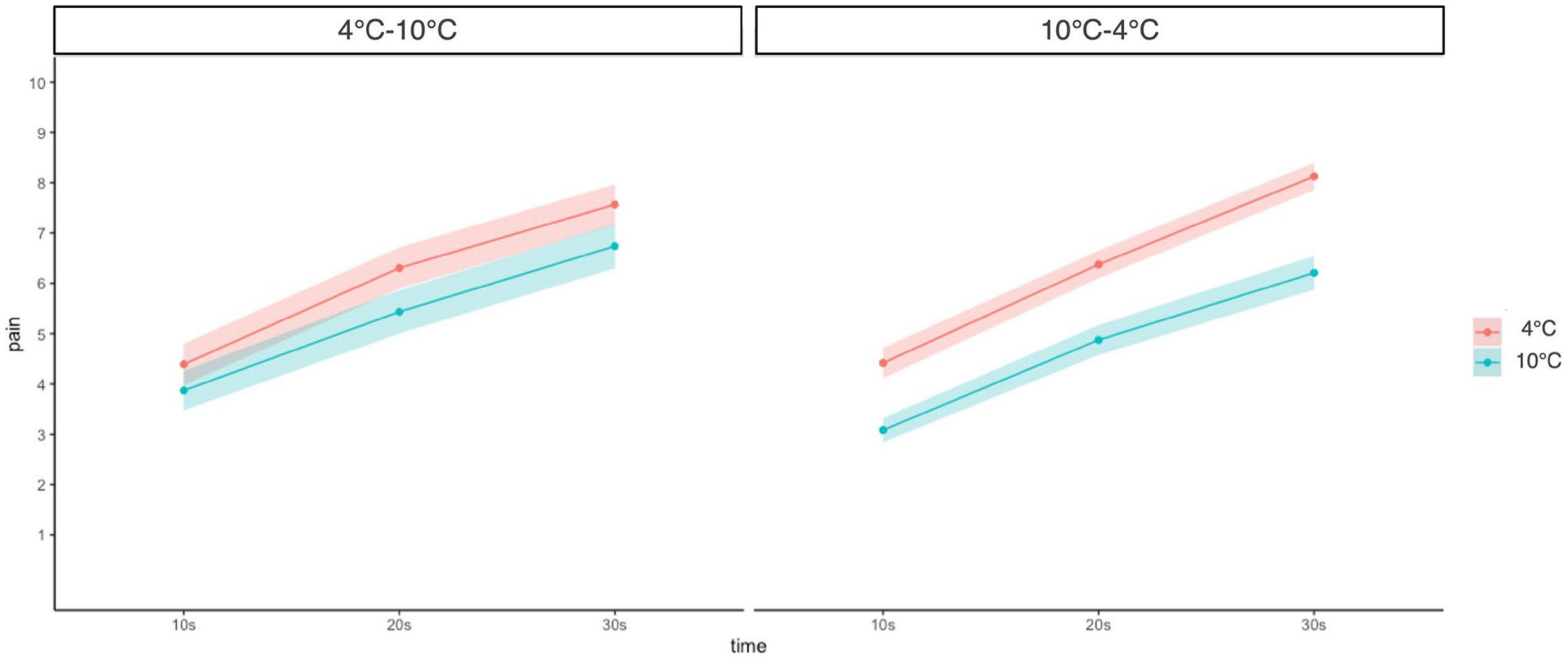
Change of pain intensity at different time points and temperatures compared between groups. Error bars represent standard errors of the mean.

A three-way (Group: 4°C–10°C and 10°C–4°C × Time: 10 s, 20 s, and 30 s × Temperature: 4°C and 10°C) mixed-measures ANOVA revealed a significant main effect of temperature (*F*(1, 45) = 79.58, *p* < 0.001, ŋ_G_^2^ = 0.11) and time (*F*(1.32, 59.61) = 248.71, *p* < 0.001, ŋ_G_^2^ = 0.38). This result indicated that pain intensity was higher in low-temperature conditions than in high-temperature conditions, and at a later time point than at the early time point in CPT. No significant interaction between group × time × temperature was found (*F*(1.75, 78.75) = 1.03, *p* = 0.36, ŋ_G_^2^ = 0.001). However, the group × temperature (*F*(1, 45) = 10.51, *p* = 0.002, ŋ_G_^2^ = 0.02) and time × temperature interaction (*F*(1.75, 78.75) = 3.79, *p* = 0.03, ŋ_G_^2^ = 0.003) showed a significant effect. Furthermore, the simple main analysis showed a simple main effect of temperature in both groups (4°C–10°C: *F*(1, 278) = 4.05, *p* = 0.045, ŋ_G_^2^ = 0.01; 10°C–4°C: *F*(1, 278) = 19.40, *p* < 0.001, ŋ_G_^2^ = 0.07). In detail, self-reported pain in the 10°C CPT was lower than that in the 4°C CPT in both groups, and this difference was higher in the 10°C–4°C group than in the 4°C–10°C group. In addition, the simple main effect of time was significant at both temperatures (4°C: *F*(2, 276) = 47.50, *p* < 0.001, ŋ_G_^2^ = 0.001; 10°C: *F*(2, 276) = 36.00, *p* < 0.001, ŋ_G_^2^ = 0.01), indicating that pain intensity increased over time at both temperatures, but the effect was higher at 10°C than at 4°C (Figure 2).

### 3.3. Pupillary response

**Figure 3:**
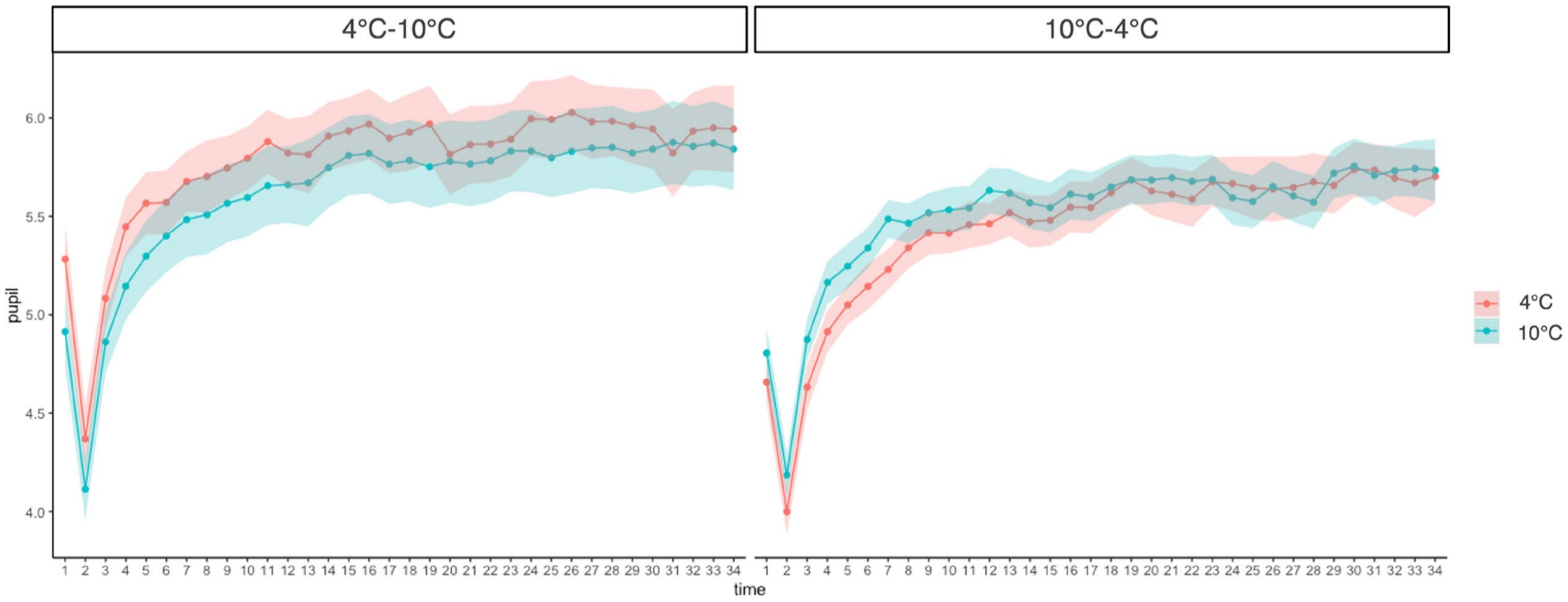
Change of pupillary response at different time points and temperatures compared between groups. Error bars represent standard errors of the mean.

A three-way (Group: 4°C–10°C, 10°C–4°C × Time: 1–35 s × Temperature: 4°C, 10°C) mixed-measures ANOVA revealed a significant main effect of time (*F*(4.59, 206.45) = 112.91, *p* < 0.001, ŋ_G_^2^ = 0.17). This result shows that as the duration of CPT increased, the pupil diameter also increased. However, there was no significant main effect of group (*F*(1, 45) = 1.24, *p* = 0.272, ŋ_G_^2^ = 0.02) or temperature (*F*(1, 45) = 0.46, *p* = 0.500, ŋ_G_^2^ = 0.001). The group × time × temperature interaction was marginally significant (*F*(6.03, 271.40) = 2.117, *p* = 0.051, ŋ_G_^2^ = 0.002) and the group × temperature interaction was also marginally significant (*F*(1.00, 45.00) = 4.03, *p* = 0.051, ŋ_G_^2^ = 0.006). Furthermore, the simple main effect of temperature was significant in the 4°C–10°C group, while it showed a marginally significant difference in the 10°C–4°C group (4°C–10°C: *F*(1, 3,192) = 13.70, *p* < 0.001, ŋ_G_^2^ = 0.004; 10°C-4°C: *F*(1, 3,192) = 3.48, *p* = 0.062, ŋ_G_^2^ = 0.001). However, the effect sizes were weak in both groups. The pupil diameter in the 4°C CPT was higher than that in the 10°C CPT in the 4°C–10°C group. In the 10°C–4°C group, the pupil diameter for the 10°C CPT was higher than that for the 4°C CPT (Figure 3).

### 3.4. Pain reappraisal

**Figure 4:**
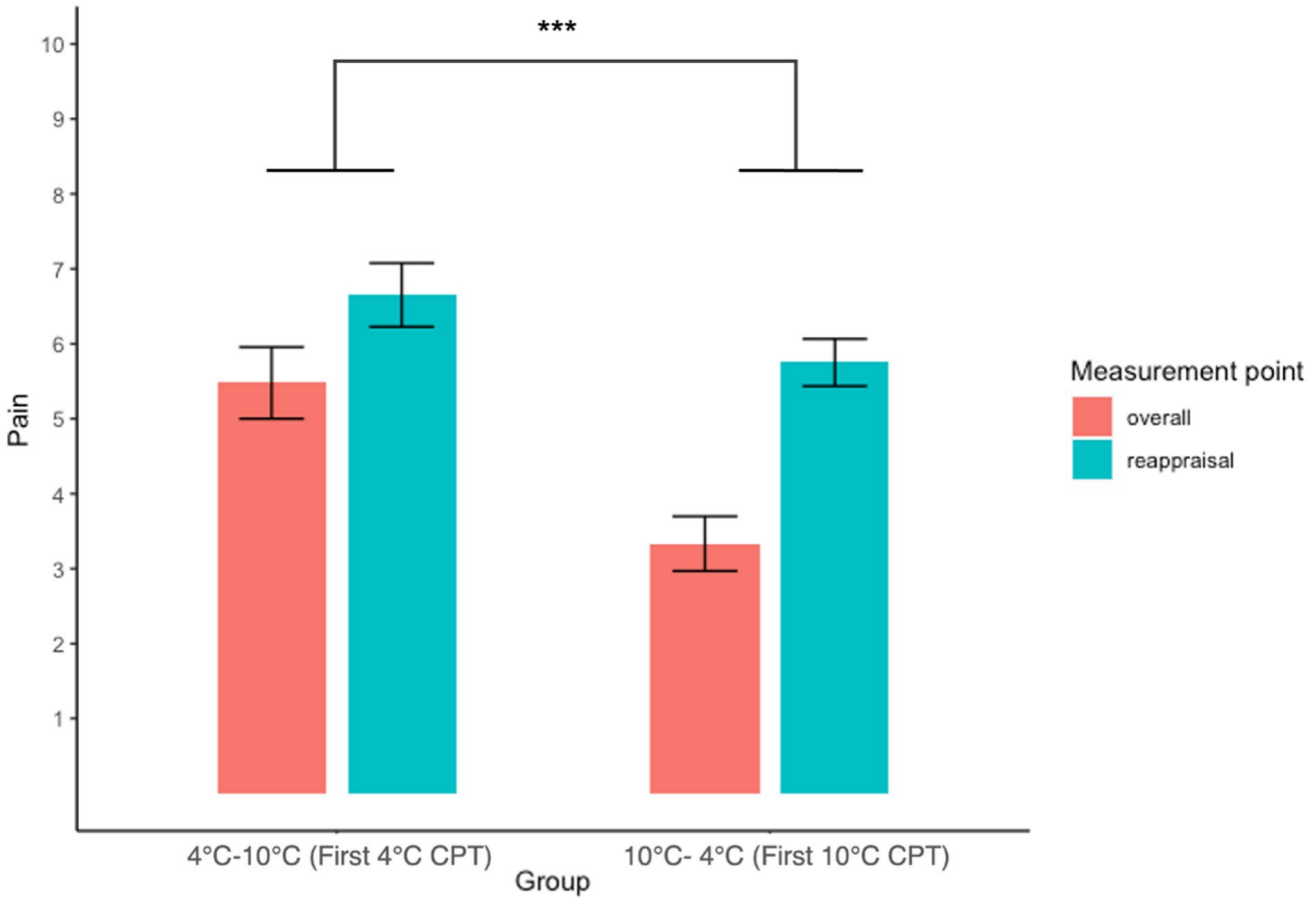
Interaction plot of overall and reappraisal with pain intensity between groups. The result of the 4°C–10°C group depicts the NRS score for the first 4°C CPT, and the 10°C–4°C group depicts the NRS score for the first 10 CPT. Error bars represent the standard error of the mean.

A two-way (Groups: 4°C–10°C, 10°C–4°C × Measurement point: overall, reappraisal) repeated measures ANOVA revealed a significant main effect of the group (*F*(1, 88) = 19.78, *p* < 0.001, ŋ_G_^2^ = 0.18), indicating that participants who had experienced 4°C CPT first rated overall and reappraised pain intensity higher than participants who had experienced 10°C CPT first. This result showed that participants reported higher pain on the 4°C CPT than on the 10°C CPT after reappraisal. There were no significant main effects of time (*F*(1, 88) = 0.59, *p* = 0.443, ŋ_G_^2^ = 0.007) and group × time interaction (*F*(1, 88) = 2.19, *p* = 0.143, ŋ_G_^2^ = 0.02). These results revealed that a recent experience of pain did not change the reappraised pain intensity (Figure 4; Table 2).

**Table 2:** Means and Standard deviations for pain intensity and pupil diameter.

	Pain (NRS)					Pupil diameter (mm)	
	10 s	20 s	30 s	Overall	Reappraisal	Mean	Max
4°C–10°C							
4°C	4.39	6.30	7.57	5.47	6.65	5.78	7.20
	(1.97)	(1.96)	(1.93)	(2.29)	(2.04)	(0.82)	(1.10)
10°C	3.87	5.43	6.74	4.74	–	5.62	6.80
	(1.89)	(2.06)	(2.12)	(2.28)		(0.94)	(1.19)
10°C–4°C							
4°C	4.42	6.38	8.13	5.25	–	5.43	6.48
	(1.47)	(1.35)	(1.33)	(1.75)		(0.57)	(0.97)
10°C	3.08	4.86	6.21	3.33	5.75	5.51	6.60
	(1.18)	(1.45)	(1.64)	(1.79)	(1.54)	(0.54)	(0.99)

## 4. Discussion

In this study, we investigated the pattern of subjective and physiological responses to cold pain stimuli and whether they were influenced by past or current pain experiences. As the limitations of self-reporting have increased, there have been numerous attempts to investigate pain responses in terms of biological parameters (Laycock and Bantel, 2016; Subramaniam et al., 2018). However, these studies have rarely considered contextual changes in pain. By understanding the influence of past and current pain experiences, we attempted to integrate the contextual perspective into pain research. Consistent with previous studies (Tassorelli et al., 1995), both self-reported pain experiences and pupil diameter increased in participants as the duration of the cold stimuli increased, regardless of temperature. We also found a main effect of temperature in self-reporting, where participants reported higher pain intensity during the 4°C CPT experiment than in the 10°C CPT experiment. Unlike self-reporting, the temperature of CPT had no significant effect on pupil diameter. Based on these results, we can assume that the subjective perception of pain is affected by both time and temperature, but that the physiological response is altered by time only.

We also confirmed that the influence of past/current pain has some differences in self-reporting and pupillary responses. Regarding self-reported pain, both groups showed lower pain intensity during the 10°C CPT than during the 4°C CPT, but the pain rating of the 10°C CPT in the 10°C–4°C group was lower than that in the 4°C–10°C group. These results suggest that, despite the same intensity of noxious stimuli, contextual factors can change self-reported pain intensity. The influence of past/current pain on self-reporting might reveal a different magnitude because the participants interpreted the NRS anchors differently. Although anchors of NRS exist (Hrvatin and Puh, 2021), the meaning of the numbers could vary according to one’s personal interpretation (Robinson-Papp et al., 2015). In particular, the label of the upper limit in NRS is prone to bias from past pain experiences because the meaning of 10 is infinite with no boundary (Walton et al., 2018). Participants in this study comprised young adults who had no history of severe pain, so the first CPT was more likely to manipulate their interpretation of the label of “worst pain imaginable.” The low intensity of the first 10°C CPT could make the 10°C–4°C group interpret the upper label of NRS as a less painful experience relative to the 4°C–10°C group, which experienced a more painful first CPT. This may contribute to a distinct perception between 10°C and 4°C cold pain stimuli by interpreting 4°C as much closer to the worst pain they can imagine than 10°C.

The results of the pupillary response revealed a different pattern from that of self-reported pain. We found that the group effect was significant in the pupillary response, indicating that the first experience of CPT alters the pupillary response in the second CPT. Contrary to our hypothesis that temperature influences pupil diameter regardless of the previous pain experience, the difference in pupil diameter was distinct, whereas the effect size was weak for both groups. It is noteworthy that the pupil diameter not only varied depending on the temperature but also on previous experiences of pain.

One possible explanation for these results is that the emotional response to the second CPT changed due to the experience of the prior CPT. Pain is usually accompanied by high emotional arousal, such as anxiety (Kapoor et al., 2016; Michaelides and Zis, 2019), pain anxiety (Cimpean and David, 2019), and fear of pain (Martinez-Calderon et al., 2019), which contribute to a worsening of the pain experience. Since the pupil dilates not only in response to nociception but also to emotional arousal (Bradley et al., 2008), emotional arousal could be the reason why the pupil dilated in different patterns. For instance, in the 4°C–10°C group, it is conceivable that participants experienced more aversive pain in their first CPT than in the 10°C–4°C group. High emotional arousal in the first pain experience may change the perception of the subsequent pain and bring out different magnitudes of pupillary response in the 4°C–10°C group by altering the emotional pain experience. Given that emotion is one of the major components of pain experience, the demand for emotion regulation may influence the pupillary response during the experience of pain. Emotion regulation can be reflected in the pupillary response because it is highly related to ANS and LC activity (Kinner et al., 2017; Ferencova et al., 2021). Previous experiences with different levels of pain may demand a different level of emotion regulation, which could lead to alterations in pupillary responses to subsequent pain.

Cognitive factors are also involved in pupillary response to pain. As the pupillary response is linked to cognitive aspects through the LC-norepinephrine system (van der Wel and van Steenbergen, 2018), pupil diameter could be affected by cognitive factors. A previous study demonstrated that the expectation of pain can alter the subsequent pupillary response (Eisenach et al., 2017). Even though the participants experienced the same intensity of 50°C, when participants anticipated lower pain (47°C), their pupil diameter was smaller than when they expected the correct intensity of pain they received (50°C) (Eisenach et al., 2017). In the current study, we also identified a similar pattern of pupil dilation; whereas the pupil diameter in the first 4°C CPT was the highest among all measurements, the pupil diameter was dilated less in the 4°C CPT after experiencing the 10°C CPT. Participants may have had a smaller pupil diameter because they anticipated less intensity of pain based on previous pain experience.

Regarding the reappraisal of the previous pain experience, self-reported pain was still higher in the 4°C CPT than in the 10°C CPT after reappraisal, even though they had a distinct recent pain experience. Although a previous study suggested that the relief of current pain alters the perception of the previous pain experience (Smith and Safer, 1993), our findings demonstrate that people can calibrate their previous pain experience accurately regardless of the current state of pain. This was in line with the results of another study, which confirmed that the rating of past pain was explained mainly by the pain intensity at the time it occurred, rather than the current pain (Daoust et al., 2017). Likewise, current pain might have had little influence on the change in the appraisal of previous pain experiences in this study. In addition, another study showed that the association between past and current pain could be weak; only a small proportion (26.5%) of individuals without pain used past pain experiences in current pain appraisal (Staud et al., 2010). Contrary to the common assumption that a patient’s report is an unreliable source of a pain experience (Marty et al., 2009; Dorfman et al., 2016), these results suggest that it is more accurate than expected, while the risk of being biased by contextual factors remains (Jensen et al., 2008; Boring et al., 2022).

These findings have several clinical implications. First, they suggest that self-reported pain scales have a potential risk of bias depending on previous pain. This implies that patients’ self-reporting of their pain could be perceived differently due to their past pain experiences. As the unreliability of a patient’s reporting of pain could lead to miscommunication between patients and clinicians and improper interventions, further investigation is needed to find solutions for these problems and address them accordingly (Seers et al., 2018). Past pain experience could be a point for adjusting the patient’s self-reporting to be more reliable. In addition, applying self-reporting pain scales with additional aids would be a way to solve related problems. For example, providing a clinician’s interpretation of self-reported pain facilitates communication easier (Bakshi et al., 2021). Training using a fixed anchor of the pain experience and a reminder card could also be beneficial for enhancing the accuracy of the pain rating (Smith et al., 2016).

Second, the pupillary response patterns identified in this study highlight the importance of proper pain management. The low intensity of previous pain leads to a decreased subsequent physiological pain response, even for the high-intensity pain stimuli. Based on this result, we can infer that relieving pain could change physiological responses to pain in the future. Likewise, previous pain experiences cause psychological symptoms and elevated pain sensitivity in the chronic pain population, whereas patients with no recent pain experience show low pain sensitivity and few psychological symptoms (Phillips et al., 2022). Given that our results underpin the influence of a previous pain experience, it also supports that timely clinical interventions that alleviate the current state of pain have not only immediate clinical benefits but also long-term benefits by preventing the exacerbation of pain and psychological problems caused by a previous pain experience.

Third, our findings support the concept of pain as a multidimensional experience, underscoring the necessity of multimodal pain assessment. Multimodal pain assessment integrates various components of pain; its benefits far outweigh single-scale pain assessment by offering a comprehensive understanding of pain (Wideman et al., 2019). One factor restricting multimodal pain assessment is the absence of valid tools to measure the various dimensions of pain (Jaaniste et al., 2019). Although some physiological parameters have been suggested, they have technical problems for use in daily practice because of inconvenient assessment equipment, such as electroencephalography (EEG) or electromyography (EMG) (Cowen et al., 2015). Pain parameters that can be assessed using easy-to-use devices are required (Wagemakers et al., 2019). The pupillary response not only reflects both physiological and psychological perspectives of pain but also provides convenience and information about real-time ANS activity (Joshi and Barreto, 2008; Maruthy et al., 2020). In this regard, integrating pupillometry into pain assessment would promote multimodal assessment and tailored treatment of pain. Our results reveal fundamental knowledge about the response pattern of the pupil and self-reporting of pain in different contexts and their contribution to the application of multimodal pain assessment.

Despite these implications, a few limitations of this study remain. First, the participants in our study consisted of a homogenous population of young, healthy Asian adults without a history of severe pain. In addition, we excluded participants who endured less than 30 s in two rounds of CPT. Therefore, our data could not report the response patterns of people with low pain tolerance. Overall, future studies should consider various demographic factors (Fillingim, 2017) and individual differences related to pain (Coghill, 2010). Second, because our results were obtained from a small sample size, further studies should validate this result with a larger sample size. Third, the study design excluded individuals with chronic pain, limiting the sample to participants with acute pain conditions. Chronic pain disorders are frequently accompanied by dysregulation of ANS activity (Tracy et al., 2016) and analgesia, which change the pupillary response (Charier et al., 2019). Further investigations are warranted to generalize our results to other patients with chronic pain. Finally, because the main aim of this study was to explore the influence of past and current pain on pain responses, we did not examine their causal relationship. Given that the subjective and physiological experiences of pain may impact each other (Caceres and Burns, 1997; Mischkowski et al., 2019), further investigations are required to identify the specific underlying mechanism and causal relationships between them.

Here, we tried to broaden our understanding of the influence of past and current pain experiences. Our findings verified that self-reported pain and physiological response to pain experience varied according to contextual differences and represented different response patterns. Through replication of this work in chronic pain conditions, it would be possible to provide a basis for applying multimodal assessment extensively and assure the prospective benefits of using pupillary response in pain assessment. In addition, this approach could contribute to effective and pragmatic pain assessments in clinical settings.

## Data availability statement

The original contributions presented in the study are included in the article/supplementary material, further inquiries can be directed to the corresponding author.

## Ethics statement

The studies involving human participants were reviewed and approved by Institute Review Board at Chungnam National University. The patients/participants provided their written informed consent to participate in this study.

## Author contributions

HY contributed to study design, data collection, organizing database, statistical analysis, and writing manuscript. YC participated in data collection and study design. SC supervised the whole process of the study and writing. All authors have contributed to the manuscript revision, read, and approved the submitted version.

## Funding

This work was supported by the Ministry of Education of the Republic of Korea and the National Research Foundation of Korea (NRF-2022S1A5A2A03050752) and Young Investigator Grant of Korean Health Psychology Association.

## Conflict of interest

The authors declare that the research was conducted in the absence of any commercial or financial relationships that could be construed as a potential conflict of interest.

## Publisher’s note

All claims expressed in this article are solely those of the authors and do not necessarily represent those of their affiliated organizations, or those of the publisher, the editors and the reviewers. Any product that may be evaluated in this article, or claim that may be made by its manufacturer, is not guaranteed or endorsed by the publisher.
